# Yeast *CUP1* protects HeLa cells against copper-induced stress

**DOI:** 10.1590/1414-431X20153848

**Published:** 2015-06-12

**Authors:** X.X. Xie, Y.F. Ma, Q.S. Wang, Z.L. Chen, R.R. Liao, Y.C. Pan

**Affiliations:** 1 Department of Animal Sciences, School of Agriculture and Biology, Shanghai Jiao Tong University, Shanghai, China; 2 Shanghai Key Laboratory of Veterinary Biotechnology, Shanghai, China; 3 College of Biological and Environmental Engineering, Zhejiang University of Technology, Hangzhou, China

**Keywords:** Yeast, Overexpression, Copper stress, Viability, ROS

## Abstract

As an essential trace element, copper can be toxic in mammalian cells when present in excess. Metallothioneins (MTs) are small, cysteine-rich proteins that avidly bind copper and thus play an important role in detoxification. Yeast*CUP1* is a member of the *MT* gene family. The aim of this study was to determine whether yeast *CUP1* could bind copper effectively and protect cells against copper stress. In this study,*CUP1* expression was determined by quantitative real-time PCR, and copper content was detected by inductively coupled plasma mass spectrometry. Production of intracellular reactive oxygen species (ROS) was evaluated using the 2',7'-dichlorofluorescein-diacetate (DCFH-DA) assay. Cellular viability was detected using the 3-(4,5-dimethylthiazol-2-yl)-2,5-diphenyltetrazolium bromide assay, and the cell cycle distribution of *CUP1* was analyzed by fluorescence-activated cell sorting. The data indicated that overexpression of yeast *CUP1* in HeLa cells played a protective role against copper-induced stress, leading to increased cellular viability (P<0.05) and decreased ROS production (P<0.05). It was also observed that overexpression of yeast *CUP1* reduced the percentage of G1 cells and increased the percentage of S cells, which suggested that it contributed to cell viability. We found that overexpression of yeast *CUP1* protected HeLa cells against copper stress. These results offer useful data to elucidate the mechanism of the *MT* gene on copper metabolism in mammalian cells.

## Introduction

Copper (Cu) is a very important intracellular trace element (1) that is required for a number of biological activities as an indispensable catalytic cofactor of many enzymes (2). However, Cu overload may initiate oxidative stress owing to redox reactions that can generate reactive oxygen species (ROS), and the accumulation of ROS will initiate oxidative damage to many biological targets (3). Metallothioneins (MTs) are ubiquitous low molecular weight peptides in eukaryotes that exhibit high Cu-binding capacity by virtue of their unusual amino acid compositions (4,5). Mammalian MTs contain a large amount (30%) of cysteine (Cys) residues, which are involved in the binding of Cu (5,6). In addition, MTs may function as intracellular antioxidants to protect cells against excessive amounts of Cu ions (3,5,7,8). MTs also play important roles in Cu homeostasis, including regulating both absorption and storage of Cu; thus they can be described as storage proteins (5).

Yeast *CUP1*, a member of the *MT* gene family, encodes a Cys-rich protein and accounts for Cu binding in the yeast *Saccharomyces cerevisiae*. The ability to bind Cu is correlated with overproduction of Cu chelation, which is determined by the number of copies of the*CUP1* gene and subsequent mRNA expression (9–11); therefore, high *CUP1* expression levels result in increased Cu-binding capacity (10,12). Phylogenetically, yeast and mammalian MTs have highly divergent primary sequences (4). However, they share identical functional sequence motifs of Cys-X-Cys or Cys-X-X-Cys, which are precisely conserved and are involved in Cu binding (9,13).

To investigate the role of a foreign MT gene on inhibition of Cu-induced stress in mammalian cells, we took advantage of the yeast *CUP1* gene for further studies. Here, the yeast *CUP1* gene was transfected into HeLa cells and a stable cell line was established. By overexpression of*CUP1*, its role in protecting cells against Cu-induced stress was evaluated. Our findings provided essential data to elucidate the role of the*MT* gene on Cu metabolism in mammalian cells.

## Material and Methods

### Cell model and viability assessment

To select the optimal Cu-His concentration, which was produced from CuSO_4_·5H_2_O and histidine (Sigma-Aldrich, USA) as described (14,15), HeLa cells were seeded onto 96-well plates at a density of 2×10^4^ cells/well. After 24 h, Cu-His at different concentrations (25, 50, 100, 200, 400, 600, 800, and 1000 μM) was added to the wells and incubated for 24 h (3). As a negative control, cells were treated with phosphate-buffered saline (PBS). The cells were washed twice with PBS to remove Cu-His, and cell viability was examined using the 3-(4,5-dimethylthiazol-2-yl)-2,5-diphenyltetrazolium bromide (MTT) reduction assay. The cells were incubated with 20 μL of MTT stock solution (5 mg/mL) at 37°C for 4 h, and 150 μL of dimethyl sulfoxide were added to formazan crystals for 20 min at room temperature. Absorbance was determined using a microplate reader (Ticen, Switzerland) at a wavelength of 490 nm. The percentage of viable cells was presented relative to the absorbance obtained from the negative control cells, which were not exposed to Cu stress, as described by Teo et al. (16).

The relative cellular viability was evaluated using the MTT assay, as described earlier, after the cells were exposed to Cu-His at a concentration of 200, 400, 600, 800, or 1000 μM for 6, 24, 48, 72, and 96 h.

### Quantification of intracellular Cu

In the following experiments, the cells stably expressing the CUP1 protein were named test cells, and the cells expressing empty vectors were used as controls. Equal concentrations of the control and test cells were seeded onto 35-mm dishes, incubated for 48 h, and then exposed for 48 h to growth medium, which was supplemented with a Cu-His complex at 10 or 100 μM. For the experiment, the cells were washed twice before Cu treatment, and the incubation medium was changed every 3 days.

After treatment, the growth medium was removed, the cells were washed twice with PBS, and then centrifuged at 8000 *g* for 5 min. Next, the cells were repelleted, dissolved in 500 μL nitric acid (Merck KGaA, Germany), and digested in boiling water for at least 2 h. After filtration, Cu content was determined by inductively coupled plasma mass spectrometry (ICP-MS; 7500 Series ICP-MS system; Agilent Technologies, Inc., USA). Each digested sample volume was standardized to 5 mL.

### Cell cycle analysis

The control and test cells, at equal concentrations, were seeded onto a 35-mm dish, incubated for 24 h, then cultured in DMEM supplemented with 0.5% fetal calf serum for 96 h to arrest cells at the G0/G1 phase (17). Then the cells were exposed to 100 µM Cu-His for 4, 8, 16, or 24 h, treated with PBS at each incubation time and used as a loading control. For cell cycle analysis, attached cells were collected, washed twice with PBS, and fixed in 70% cold ethanol at 4°C for 24 h. After fixation, ethanol was removed and propidium iodide (PI) buffer (20 μg/mL of RNase A and 20 μg/mL of PI in PBS; Sigma-Aldrich) was added. After 30 min of incubation, the cell cycle profile was analyzed using a FACSCalibur (Becton Dickinson and Company, USA). Data were collected from at least 10,000 fluorescent cells per sample and analyzed using Coulter System software (Becton Dickinson and Company).

### Detection of intracellular ROS

The control and test cells grown on 35-mm dishes were treated with Cu-His at 200, 400, 600, 800, or 1000 μM for 48 h, and the production of intracellular ROS was evaluated using the DCFH-DA (2',7'-dichlorofluorescein-diacetate) assay (18). After treatment, the cells were incubated with DCFH-DA probes for 30 min, then washed twice with PBS. Dichlorofluorescein (DCF) fluorescence was read at an excitation wavelength of 485 nm and emission wavelength of 528 nm using a fluorescence microplate reader (Bio-TEK Instuments, Inc., USA).

### Statistical analysis

Variables of at least three separate experiments were tested and the results are reported as means±SE. Variable differences were compared using the*t-*test and analysis of variance using the SPSS version 16.0 statistical software (USA). P<0.05 was considered to be significant.

## Results

### Concentrations of Cu-His

**Figure 1:**
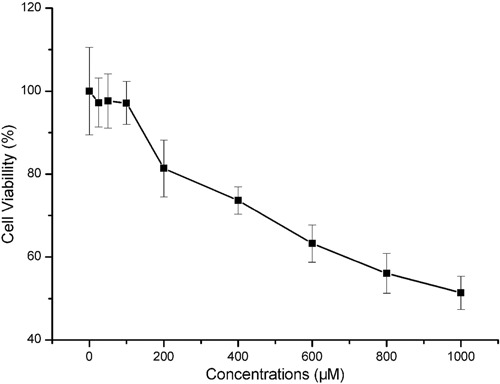
Percentage of viable of HeLa cells at different Cu-His concentrations. Cellular viability was analyzed using the MTT assay. Cu-His at different concentrations (200, 400, 600, 800, and 1000 μM) inhibited cell viability by approximately 20%-50% (P<0.01,*t*-test), but not for concentrations under 100 μM (n=8) (P>0.05). The results were reported relative to the response of the negative control cells. MTT, 3-(4,5-dimethylthiazol-2-yl)-2,5-diphenyltetrazolium bromide.

As shown in Figure 1, Cu-His effectively inhibited the cytoactivity of HeLa cells with an obvious loss of approximately 20–50% cell viability when Cu-His was introduced into the cells at different concentrations (200, 400, 600, 800, or 1000 μM), indicating that the cells were under Cu stress, and Cu-His at concentrations under 100 μM was not cytotoxic to the cells. No obvious dead cells were observed when Cu-His was at the highest concentration of 1000 μM. The concentrations over 100 μM were used for further experiments on Cu stress.

### Intracellular Cu content

To investigate whether Cu binding was highly correlated with*CUP1* mRNA and protein levels, intracellular Cu content was analyzed. The results indicated that Cu content in the test cells was significantly greater than that in the control cells at both concentrations of Cu-His (P<0.01), and the difference increased when Cu-His concentrations were increased from 10 to 100 μM (Table 1). The results indicated that yeast *CUP1*overexpression could bind Cu effectively in HeLa cells and increased intracellular Cu content.

**Table:**
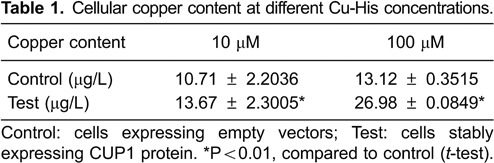

### Cell viability analysis

**Figure 2:**
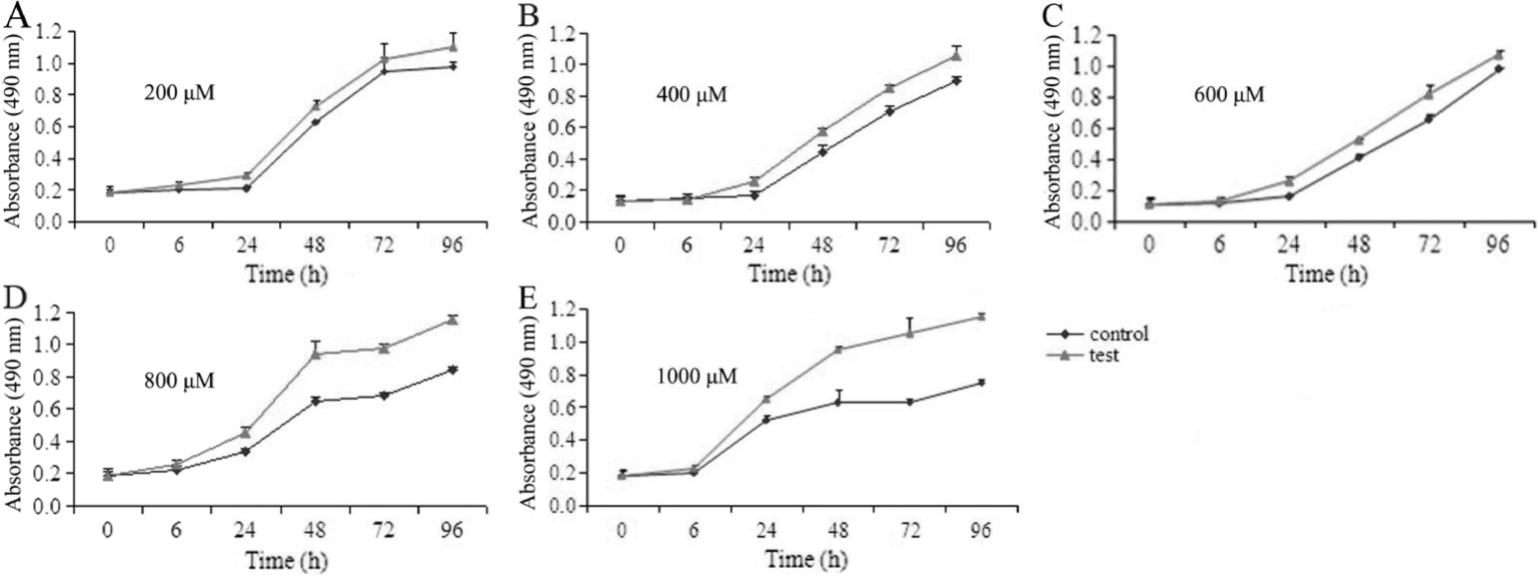
Cellular viability assay results of the control and test cells. The capacity for cellular viability was examined using the MTT assay and cellular viability was compared between the control and test cells (n=8). *A*-*C*, The cellular viability of the test cells was significantly greater than that of the control cells (P<0.05) exposed to Cu-His at 200, 400, and 600 μM;*D*,*E*, the differences were significant (P<0.01) among cells exposed to Cu-His at 800 and 1000 μM. The *t*-test was used for statistical analysis.

After incubation, cell viability was analyzed using the MTT assay to compare the average absorbance of the test cells with that of the control cells. A comparison of the relative viability of the cells after treatment with Cu-His at different concentrations is shown in Figure 2, A-E. The results demonstrated that the viability of the test cells was significantly greater than that of the control cells (P<0.05) after treatment with Cu-His at 200, 400, and 600 μM (Figure 2, A-C), and the differences were also significant (P<0.01) after treatment with Cu-His at 800 and 1000 μM (Figure 2, D and E). Comparatively, the test cells appeared to have a greater viability at all incubation times, supporting a protective role against excess Cu. Hence, yeast*CUP1* may allow the cells to bind more Cu, resulting in an increase in the intracellular antioxidative ability to protect the cells against excessive amounts of Cu, as reported by Richards (7).

### CUP1-mediated cell cycle

Based on the above results, using FACS we further investigated whether the cell cycle was mediated by yeast *CUP1*. Cell cycle analysis showed a high level of cycle synchronization, and the cells were mostly arrested at G0/G1 phase after 96 h of serum starvation. However, a decreased proportion of cells was in the G1 phase (P<0.01) and an increased proportion of the test cells was in the S phase (P<0.01) relative to the control cells when incubated with Cu-His at 100 μM for 4, 8, 16, and 24 h (Table 2), but no significant difference was observed between HeLa and control cells (P>0.01). The same was also observed between the HeLa cells, control and test cells when incubated with PBS for all incubation times (P>0.01; Table 3).

**Table:**
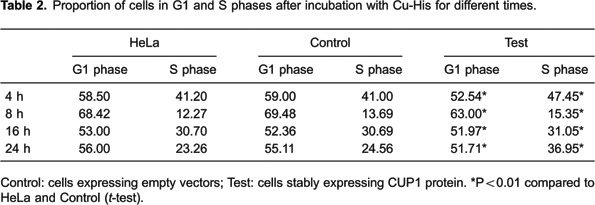

**Table:**
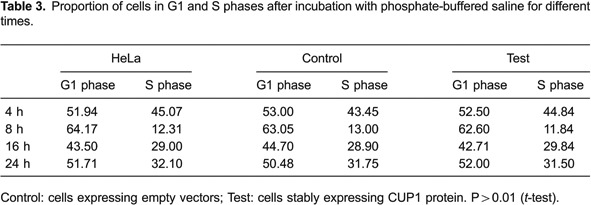

### Intracellular ROS

**Figure 3:**
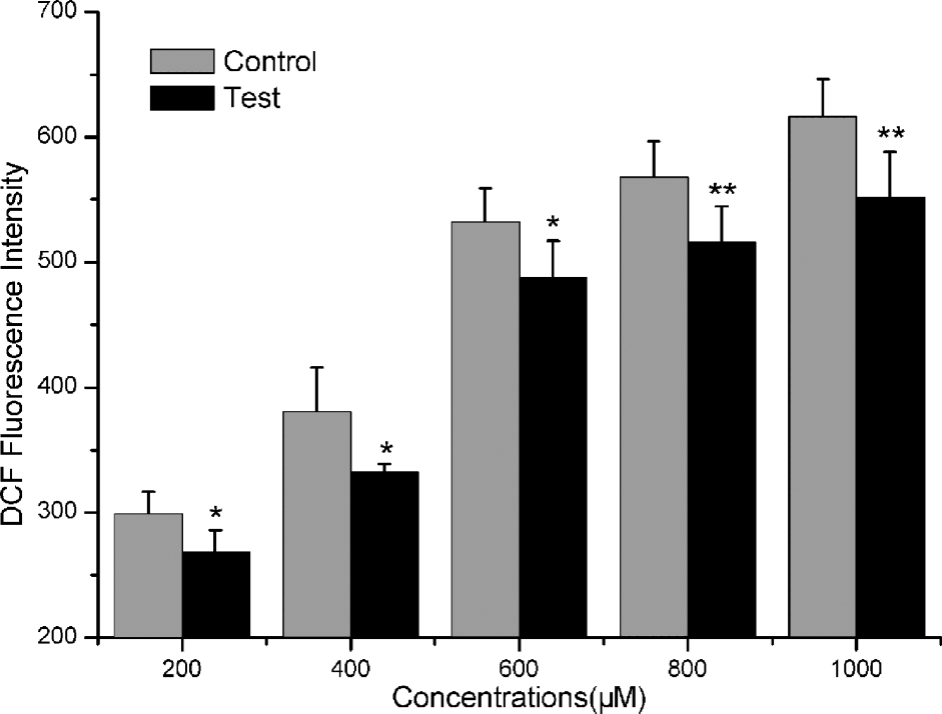
Effects of yeast *CUP1* on intracellular ROS. ROS formation, which was determined by fluorescence intensity, was detected as the measurement of copper stress. Data are reported as means±SE (n=8). ROS: reactive oxygen species.*P<0.05,**P<0.01, compared to control cells (*t*-test).

Considering the damage to the cells upon treatment over the range of high Cu concentrations, we detected ROS production as a measurement of Cu stress using the DCFH-DA assay. An increase in fluorescence intensity indicated an increase in intracellular ROS (18). The fluorescence intensity of the test cells was significantly lower than that of the control cells (P<0.05) after treatment with Cu-His at 200, 400, and 600 μM, and the differences were also significant (P<0.01) after treatment with Cu-His at 800 and 1000 μM (Figure 3).

## Discussion

The functions of MTs, such as storage of metal ions, metal detoxification, and oxidative scavenging, have been extensively studied (19), but the roles of MTs on intracellular antioxidant activity remain elusive. In the present study, our goal was to elucidate the role of yeast*CUP1* in Cu metabolism, as well as its functions on cellular Cu content, cell viability, cell cycling, and intracellular ROS. Cell lines that stably expressed yeast *CUP1* were used to assess whether yeast*CUP1* can bind Cu effectively and protect cells against Cu stress.

Our findings indicated that the expression of yeast *CUP1* was highly abundant in HeLa cells (Supplementary Figure S1). CUP1 possesses identical Cu-binding geometry with human MT (4), as shown in the HeLa cells. In the presence of Cu (100 μM for different durations), the relative abundance of human MT increased with incubation times, in accordance with previous observations (15,19), whereas no increase in*CUP1* mRNA expression was observed (Supplementary Figure S2), because *CUP1* expression was initiated by the cytomegalovirus promoter of the pEGFP-N1 plasmid. Our results indicated that *MT*plays an important role in the Cu-dependent induction of its own transcription, which was in agreement with the results of previous studies (15,20). At all incubation time points, expression of *CUP1* mRNA was significantly greater than that of human *MT* mRNA, suggesting that*CUP1* played a dominant role in binding Cu compared to the human*MT* gene.

MT is a primary Cu-binding protein under physiological conditions (21), and characterization of the MT-Cu complex suggests that MT is beneficial for intracellular storage of Cu (15). It has been demonstrated that an increase in the content of cellular Cu is directly correlated with an increase in the amount of MT-Cu (22), and MT was involved in the process of Cu absorption and storage (5,19). In our experiments, the increase in cellular Cu content resulting from overexpression of yeast *CUP1*demonstrated that *CUP1* possessed capabilities of cellular storage within the physiological range of Cu exposure. Additional evidence has shown that different cells exhibit increased Cu content in response to a gradual increase in Cu exposure (19,23), and a similar phenomenon was observed in our experiments.

Cu is a very important catalytic cofactor in many biological processes (1), and Cu deficiency compromises cellular antioxidant defense capability, thereby increasing cellular susceptibility to oxidative DNA damage (24). However, enhanced Cu can lead to cytotoxicity due to ROS formation (1). High levels of exogenous ROS directly inactivate protein phosphorylation and interfere with the balance of cellular kinase/phosphatase activity toward added enzymatic phosphorylation events (25). Some nutrients reportedly provide protection against Cu-induced oxidative damage by acting as nonenzymatic antioxidants, such as vitamin C, vitamin E, and glutathione (26). Cu/Zn superoxide dismutase (SOD) and catalase are enzymes that efficiently eliminate ROS by catalyzing the breakdown of excess superoxide and H_2_O_2_, and are involved in antioxidant defense (25). Upregulation of SOD and catalase expression leads to reduced ROS levels (27), which, in turn, seems to promote cellular viability, whereas increased ROS generation can suppress cellular activity by inhibiting activities of SOD and catalase, which protect cells against oxidative stress through the dismutation of superoxide to O_2_ and H_2_O_2_ (27,28). Reducing oxidative stress by nonenzymatic antioxidants as well as antioxidant enzymes could potentially reduce ROS formation (29). Our findings indicated that overexpression of yeast *CUP1* resulted in decreased intracellular ROS formation, which supports a protective role for MT (CUP1) in response to Cu excess by inhibiting ROS formation as nonenzymatic antioxidants, similar to the findings of Tapia et al. (15).

It has been strongly suggested that MT protein content is directly associated with resistance to excess Cu exposure in mammalian cells (19,23), which protects against Cu-dependent cytotoxicity by its antioxidant activity (30) and could eliminate ROS generated from Cu exposure (19), or primarily by its ability to bind Cu with high affinity. Thus, the multiple Cys residues in MT act as effective Cu chelators that react with ROS and can effectively protect the cell from Cu toxicity (31). Conditions correlated with Cu overload may lead to Cu-induced stress (19), which gives rise to the production of increased amounts of ROS capable of generating oxidative stress, because Cu can function as a transition metal with redox cycling capacity (20). Here, the results of the MTT assay showed an increase in viability of the test cells compared to the control cells. Because of the close relationship between cell viability and the cell cycle (32), the cell cycle was further analyzed. Thus, the decreased proportions of G1 phase cells and the increased proportions of S phase cells suggest enhanced cellular viability (33,34). One reasonable explanation for this observation is the abundance of yeast*CUP1* produced in the test cells that likely bound the Cu, which stimulated an increase in cell viability, perhaps by ameliorating oxidative stress or reducing ROS production (1), because viability in cells lacking Cu/Zn-SOD can be complemented by *MT*overexpression (28). In summary, our study provided essential insights into the physiological regulation of yeast*CUP1* on binding Cu and blocking Cu-induced stress. We found that overexpression of yeast *CUP1* was beneficial to protect HeLa cells against Cu stress.

## Supplementary Material

Click here to view[pdf].
